# Demand-oriented regionalization with local data

**DOI:** 10.1038/s41598-026-47764-4

**Published:** 2026-04-20

**Authors:** Seyedeh Mobina Noorani, Shangde Gao, Changjie Chen, Karla Saldaña Ochoa

**Affiliations:** 1 College of Design, Construction and Planning, University of Florida, Gainesville, 32611 USA; 2 Department of Electrical and Computer Engineering, University of Florida, Gainesville, 32611 USA; 3 Florida Institute for Built Environment Resilience (FIBER), University of Florida, Gainesville, 32611 USA

**Keywords:** community resilience, Heterogeneous data, Planning support, Regionalization, Self-organizing map, urban governance, Environmental sciences, Environmental social sciences, Mathematics and computing

## Abstract

Climate adaptation planning often relies on census-based or neighborhood boundaries, yet such fixed units seldom match the diverse and evolving problems that interventions seek to resolve. This underscores the necessity of designing demand-oriented regionalization that is problem-specific and responsive to local priorities. Traditional regionalization methods, however, struggle to balance socioeconomic and environmental variables while maintaining spatial coherence to meet practical and physical constraints. To address these shortcomings, we present ***RepSC-SOM***, a **Rep**resentative-initialized, **S**patially **C**onstrained **S**elf-**O**rganizing **M**ap that extends traditional SOM with representative-based initialization, adaptive geographic filtering, and region-growing refinement. The method maximizes within-region similarity and between-region dissimilarity while maintaining spatial coherence. Notably, the framework is designed to enhance transparency and interpretability by standardizing how planning regions are defined, reducing reliance on subjective or historically bounded spatial units. Applied to flood-induced water contamination (E. coli concentrations) in Jacksonville, FL, RepSC-SOM-generated regions provided a more accurate characterization of the problem than standard units of analysis. Specifically, these regions achieved a higher average pairwise difference (223 per 100mL) compared to census tracts (154 per 100mL, $p=0.002,r=0.088$), traffic analysis zones (118 per 100mL, $p<0.001, r=0.154$), and neighborhoods (145 per 100mL, $p=0.001,r=0.101$), indicating accurate detection and more precise delineation of contamination hot spots. These results suggest a strong potential for applying RepSC-SOM in real planning contexts to guide targeted interventions, prioritize resource allocation, and support coordinated climate adaptation strategies.

## Introduction

Urban governance relies on designated planning units (or ***regions***) to design and implement policies for growth, management, and adaptation to socioeconomic and environmental changes^1^. As a spatial framework, such regions allow planners to evaluate resource distribution^2^, assess policy impacts^3^, and ensure coherence of policy implementation across multiple scales^4^. They also serve as a reference to facilitate stakeholder engagement^5,6^, and support decision-making.

Standard planning units—such as administrative boundaries, census divisions, or regions established for specific purposes—are widely adopted to solve problems in cities^7^. However, since most existing planning units were originally designed for specific tasks, they may not align with the requirements of specific planning demands. For example, census units (e.g., blocks, block groups, tracts) are defined for population-related data collection and estimation^8,9^ and, therefore, do not reflect hazard-exposure patterns or social vulnerability. Similarly, the spatial distribution of climate risks may not be highlighted by other standard planning units^10^. Also, a key challenge in spatial analysis is the Modifiable Areal Unit Problem (MAUP), whereby analytical results can vary depending on how spatial boundaries are defined^12,33^. Consequently, reliance on administrative units for environmental analysis is inherently subject to scale and zoning effects, which may obscure or distort underlying spatial patterns.

The potential mismatch between standard planning units and the demands of urban governance underscores a ***demand-oriented regionalization*** to meet specific planning purposes, but nonetheless, several challenges continue to hinder its implementation. On the one hand, performing regionalization, or redistricting, is often politically and administratively complex^13^. The boundaries of standard planning units are typically negotiated through lengthy processes, reflecting the balance of interests among stakeholders, historical heritage, and administrative convenience^14^. Once institutionalized, planning units can be embedded in laws, governance structures, and funding mechanisms^7^, creating strong resistance to modification. Consequently, even when urban dynamics or environmental conditions change, standard planning units can remain rigid, restricting the flexibility for certain planning demands. On the other hand, local contexts include fine-grained social, economic, and environmental dynamics^15^ that can be captured primarily through high-quality local data^16^, but these data are usually scarce, inconsistent, or inaccessible^17^. However, without close engagement with sufficient knowledge and reliable data on local contexts, designed planning units can risk being misaligned with on-the-ground realities, reducing their relevance and legitimacy.

Data-driven regionalization offers a promising solution to these challenges by integrating multidimensional local data and expert knowledge to create regions that are relevant to planning objectives and adaptable to practical constraints^18–20^. Typically formulated as a clustering process, data-driven regionalization can generate spatially contiguous and homogeneous regions by solving a constrained optimization problem that maximizes within-region similarity and between-region dissimilarity under spatial constraints^18^. Unlike the delineation of standard planning units, data-driven regionalization produces spatial units that reflect shared characteristics across social, environmental, and physical dimensions^19^. These regions can capture the spatial heterogeneity of climate risk and support evidence-based decision-making.

Today, urban areas face various disasters and climate risks driven by distinctive environmental and socioeconomic conditions^21,22^. For example, flooding has caused extensive damage across the coastal Southeastern U.S.^23^, while metropolitan centers like Los Angeles are increasingly exposed to extreme heat^24,25^. These diverse threats underscore the need for planners to design localized strategies to mitigate the risks posed by weather-related hazards^22^. Data-driven regionalization can support targeted adaptation planning by identifying priority areas for resource allocation and intervention, but its application for this purpose remains limited, and a comprehensive method for data-driven regionalization remains lacking.

In this study, we propose a novel framework called ***RepSC-SOM*** (**Rep**resentative-initialized, **S**patially **C**onstrained **S**elf-**O**rganizing **M**ap) to generate demand-oriented regions by integrating multi-dimensional local data and spatial constraints, enabling more customizable and data-driven urban governance. RepSC-SOM addresses the challenges of demand-oriented regionalization by extending the traditional SOM model with representative-based initialization, adaptive geographic filtering, and region-growing refinement. Driven by the idea of integrating high-quality, local socioeconomic and environmental data, RepSC-SOM generates spatially coherent regions that capture local vulnerabilities and complex hazard-risk profiles, overcoming the rigidity and misalignment of standard planning units. This framework can also increase the flexibility in adapting to changing urban dynamics and environmental conditions, while ensuring that regions reflect the realities of local contexts.

## Related works

**Regionalization** has been applied in many fields, such as environmental management^26^, land use planning^27^, transportation^28^, public health^29^, and climate zoning^30^, which defines spatial units that reflect feature similarities. For example, regionalization supports planning for ecological systems by generating regions based on local vegetation, climate conditions, and land cover, balancing the demands of urban development and biodiversity preservation^18^. Researchers also use regionalization to support transportation planning by characterizing travel flow distributions on holidays and during normal workdays^31^. In public health, it aids in clustering areas with similar disease prevalence to support targeted interventions and protect privacy data^29^. These applications typically aim to improve analytical precision, optimize resource allocation, or support decision-making within domain-specific contexts^18,27^. Despite its versatility, regionalization remains underutilized in resilience and adaptation planning. The insufficient integration of multidimensional spatial data, which captures hazard exposure and local vulnerability, limits planners from defining regions that are both actionable and reflective of the complex climate and disaster risk drivers.

**Traditional methods for regionalization** that require spatial contiguity include: 1) optimization models, 2) heuristic strategies, and 3) a combination of both^18,32^. Optimization-based regionalization methods, such as the Automatic Zoning Procedure^33^ and AZPTabu^34^, formulate regionalization as a combinatorial optimization problem, seeking to partition spatial units into contiguous, homogeneous regions that optimize a given objective function, often related to within-region similarity or between-region heterogeneity. To efficiently explore the solution space and enforce spatial contiguity constraints^34^, these methods typically employ metaheuristics, such as simulated annealing^35^ or tabu search^36^. In contrast, heuristic and graph-based models, such as SKATER^37^ and REDCAP^38^, construct regions by iteratively merging or splitting spatial units based on attribute similarity and adjacency, often utilizing minimum spanning trees or hierarchical clustering to ensure spatial coherence. Other approaches, such as the P-Regions problem^39^, construct regions based on user-defined constraints on the maximum number of homogeneous regions, while ensuring that the value of a spatially extensive regional attribute exceeds a predefined threshold value.

**Self-Organizing Maps (SOM)**^40^ is a type of unsupervised neural network that projects high-dimensional input data onto a lower-dimensional (typically two) grid of neurons, preserving the topological and similarity relationships among data points. In the context of regionalization, SOM enables the identification of regions by grouping spatial units with similar multivariate characteristics, such as socioeconomic or environmental attributes^29,41^. Each neuron on the SOM grid represents a prototype vector^42^, and spatial units are assigned to the neuron whose prototype is most similar to their feature vector, effectively partitioning the study area into homogeneous regions, though without guaranteeing spatial contiguity. As an artificial intelligence (AI) method, SOM provides an interpretable two-dimensional representation of complex data, enhancing transparency by enabling planners to visualize and trace how clusters and regions are formed^43^. This interpretability supports more informed and accountable decision-making; however, SOM does not explicitly enforce spatial contiguity, which may lead to fragmented or irregular regions. This limitation motivated the development of Geo-SOM, which incorporates geographic constraints into the SOM framework^44,45^.

**Geo-SOM** has been proposed for understanding the spatial patterns of georeference data and to advance SOM and explicitly incorporate geographic information—such as spatial coordinates or geographic adjacency—into the clustering process^44,45^. During training, Geo-SOM modifies the neighborhood function or the distance metric to account for both attribute similarity and spatial proximity, ensuring that the resulting regions are not only homogeneous in feature space, but also spatially contiguous and geographically meaningful^44,46^. This spatial constraint helps prevent the formation of fragmented or scattered regions, which is critical for practical applications in urban planning and disaster management^47,48^. Geo-SOM has been applied to regionalization tasks, including delineating health service areas^29^, zoning social interactions^49^, and defining regions for hazard risks^50^, demonstrating its ability to generate interpretable and operationally relevant spatial partitions.

However, the effectiveness of Geo-SOM for regionalization can be constrained by several design limitations. First, Geo-SOM random weight initialization can overlook the spatial distribution of the input data^44^, leading to slow convergence, misalignment with spatial patterns, or suboptimal region formation. Second, Geo-SOM uses a fixed grid-based radius when assigning areas to BMUs^29,46^, limiting its ability to adapt to irregular spatial configurations. This issue may potentially result in fragmented regions in dense areas and large heterogeneous regions in sparse areas, both of which are not suitable for planning practices^51^. Third, Geo-SOM may lack post-processing steps that are commonly used to enforce spatial connectivity and compactness^18^, further increasing the risk of generating fragmented or irregular regions.

In this study, we propose RepSC-SOM, a fully data-driven regionalization framework that addresses the limitations of Geo-SOM outlined above. Specifically, RepSC-SOM derives spatial initialization directly from the intrinsic spatial patterns of the data, avoiding arbitrary starting conditions. It also adapts neighborhood structures to local variations, enabling the method to capture fine-scale heterogeneity in the climate and disaster risk. Additionally, RepSC-SOM integrates a region-growing process that enforces spatial contiguity and compactness, producing regionalization outputs that are geographically coherent and operationally suitable for planning and decision-making.

## Results

### Overview of RepSC-SOM framework

**Fig. 1:**
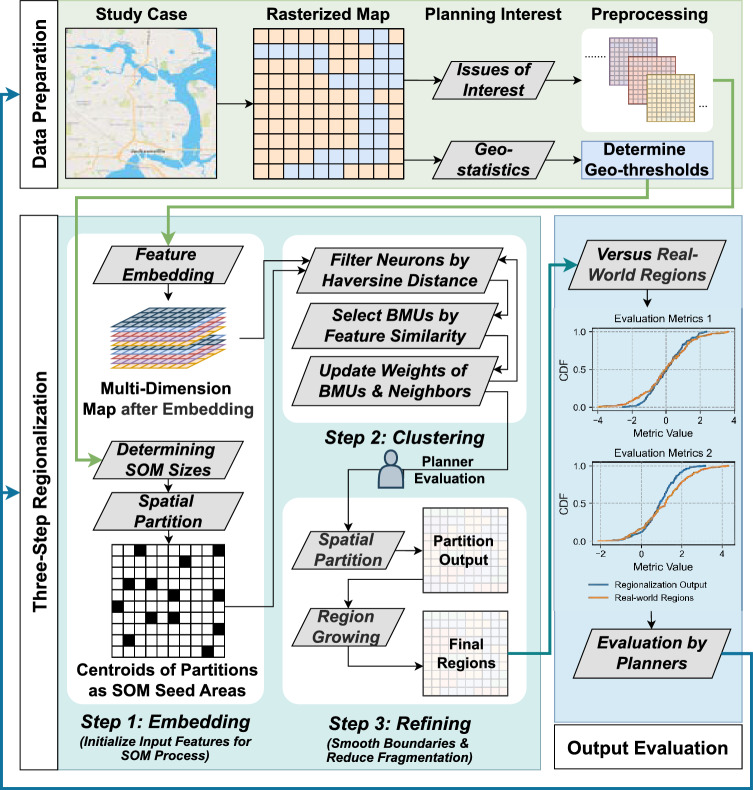
Overview of the RepSC-SOM framework, which includes three stages: **Data Preparation**, where the study area, issue of interest, input features, and geo-thresholds are defined; **Three-step regionalization**, in which the ***Embedding*** step projects input features into a latent space via an autoencoder, the ***Clustering*** step assigns grid cells to Best Matching Units (BMU) considering spatial neighbors, and the ***Refining*** step merges preliminary regions to improve spatial coherence; and ***Output Evaluation***, where the resulting regions are compared with real-world delineations (e.g., census tracts, TAZ, neighborhoods) to assess their ability to capture spatial patterns of climate adaptation outcomes, such as water contamination levels in this study. Planners can be involved in every step and determine if the output from that step can be delivered to the next step.

The proposed RepSC-SOM framework (Fig. 1) consists of three stages: data preparation, three-step regionalization, and regionalization evaluation (see Methods for details). The outputs at each stage are transparent and interpretable, enabling users to trace how regional boundaries emerge from input data, model parameters, and intermediate results. Also, the systematic workflow of selecting and calibrating parameters through sensitivity analysis and domain-informed evaluation is transferable to other urban contexts, enabling researchers to adapt the RepSC-SOM framework to their specific study areas and research questions.

In the first stage, ***Data Preparation***, users define the study area (e.g., a city) and the issue of interest (e.g., flooding-induced water contamination), and select input features aligned with the planning objective. The geographic threshold (geo-threshold) for spatially constrained SOM regionalization is then automatically determined from the geostatistics of the input data.

Then, regionalization is performed using a SOM-based three-step ***Embedding–Clustering–Refining*** process (see Methods for details), in which the study area is represented as a raster of grid cells. In the ***Embedding*** step, input features are projected into higher-dimensional latent spaces using an autoencoder^52^, capturing complex interactions and dependencies among variables. At the same time, the SOM is initialized according to the predetermined geo-threshold, which is the basis for both the number of neurons and the initial states of the neurons. The ***Clustering*** step then uses the embedded features as inputs to the spatially constrained SOM, iteratively assigning grid cells to their Best Matching Units (BMUs) and updating the weights of the BMU and its spatial neighbors, thereby forming preliminary clusters. In this process, the selection of the BMU for each grid cell involves two stages: candidate SOM neurons are first identified within a geo-threshold using the Haversine distance^53^, and then the most similar SOM neuron in the feature space is chosen as the BMU. During training, the weight vectors of the BMU and its neighbors are incrementally adjusted toward the features of the input grid cells. After multiple iterations, each grid cell is associated with the BMU whose weight vector best represents its features. Finally, in the ***Refining*** step, the spatially constrained SOM output is post-processed to improve spatial compactness and reduce fragmentation: these BMU-labeled grid cells are first spatially partitioned into initial regions, which are then iteratively merged through a region-growing process guided by feature similarity and spatial constraints.

In the third stage, ***Output Evaluation***, the regionalization results are compared with real-world delimitations (e.g., census tracts, traffic analysis zones, neighborhoods) based on their ability to capture spatial patterns in climate adaptation outcomes, exemplified here by regional variations in water contamination levels. The evaluation process compares the statistical distributions of cross-regional differences in climate adaptation outcomes between our framework outputs and real-world delineations.

### Case description and input features

We apply the RepSC-SOM framework to Jacksonville, Florida, an urban area facing heightened risks of flooding and significant vulnerability of the infrastructure under intensifying climate change^54^. Our case study focuses on supporting targeted governance strategies to mitigate the risks of waterborne diseases^55^, which can be exacerbated by decentralized wastewater systems (e.g., septic tanks) during flood events^56^. The water quality data used for validation were obtained from the STORET and WIN databases of the Florida Department of Environmental Protection^57,58^, with contributions from multiple agencies, including the National Park Service, City of Jacksonville, and St. Johns River Water Management District. The dataset spans from November 2016 to September 2024, with the majority of observations collected between 2017 and 2023. This multi-year temporal coverage and multi-agency sourcing help reduce the influence of short-term temporal variability and individual measurement uncertainty, providing a more robust basis for validating the regionalization output.

**Fig. 2:**
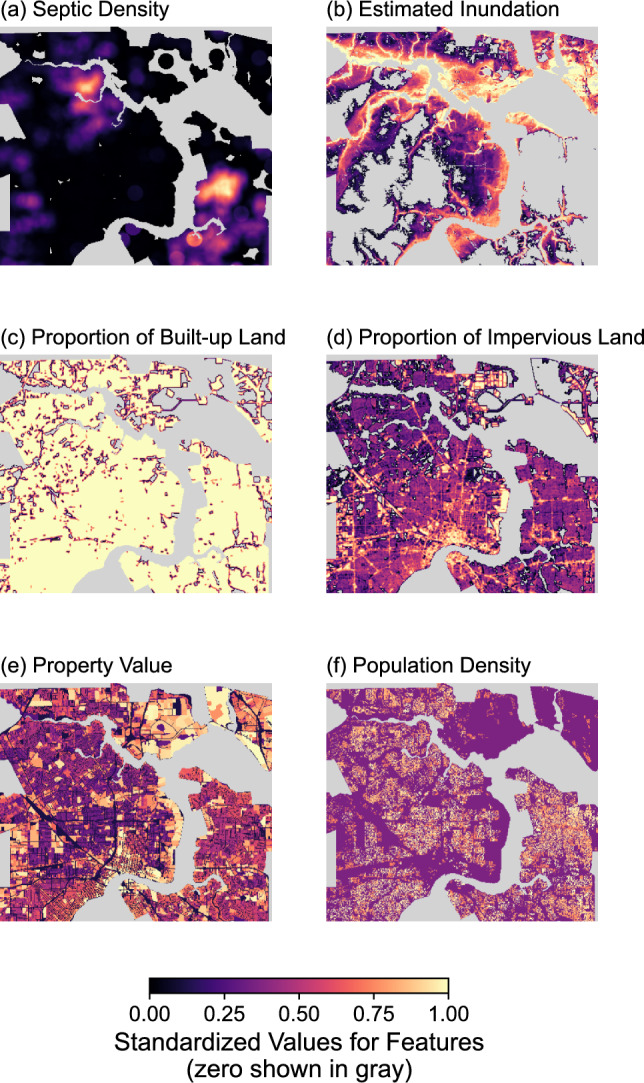
Input features used in the RepSC-SOM regionalization: (**a**) density of septic tanks per grid cell, representing sanitation vulnerability during floods^62^; (**b**) estimated inundation level (meters above ground level, m AGL) under a Category 5 hurricane^61^; (**c**) proportion of built-up and transportation areas^64^; (**d**) proportion of impervious land within each grid cell^65^; (**e**) percentile of within-cell average property values; (**f**) population density based on Meta Data for Good^66^.

Six input features were selected for RepSC-SOM (Fig. 2): the count of septic tanks, expected flooding inundation level, the percentile of property values across the study area, the proportion of built-up areas, the proportion of impervious land, and population density (described in **Data Preparation** subsection of **Methods**). The count of septic tanks reflects the dependence on decentralized wastewater facilities, whose failure can affect pathogen exposure during flooding. The expected inundation levels capture the flood risk driven by sea level rise and extreme weather events (e.g., Category 5 hurricanes). Property values and population density serve as a proxy for socioeconomic capacity and potential disparities in adaptive resources. The proportion of built-up areas and impervious land can influence flood impacts by affecting water flow and surface permeability. Together, these input features can cover key dimensions of urban governance under climate stress–linking infrastructure, environmental risk, and social equity.

### Regionalization with RepSC-SOM in the case area

**Fig. 3:**
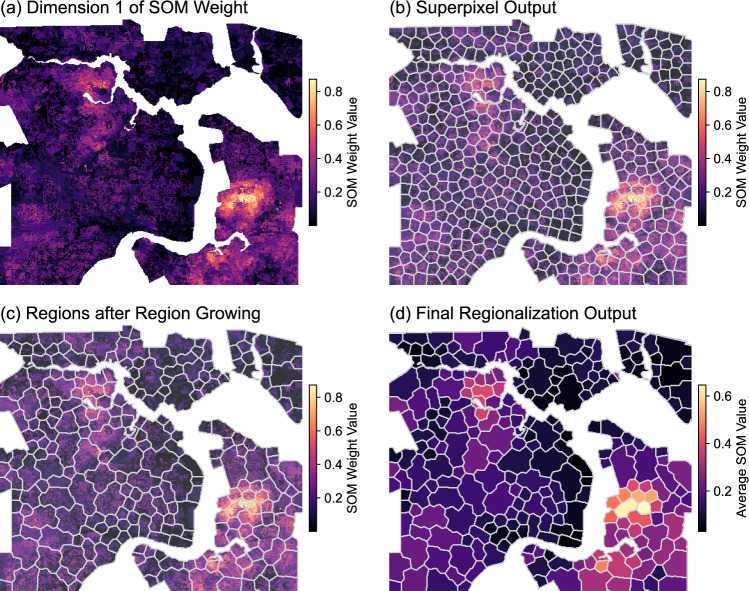
Mapping of outputs during the ***Clustering*** and ***Refining*** steps of the regionalization process. The ***Clustering*** step produces a multi-dimensional map, where each dimension of a grid cell (e.g., Dimension 1 in subfigure (**a**)) corresponds to the SOM weight for the embedded feature. This map is then processed through spatial partitioning (e.g., superpixels, subfigure (**b**)) and region-growing refinement (subfigure (**c**)), resulting in the final regionalization output shown in subfigure (**d**).

Following the process of RepSC-SOM (Fig. 1), we first decided on the data resolution in the case area as $50\,\textrm{m} \times 50\,\textrm{m}$, a scale selected to align with the neighborhood-level focus of our research question – fine enough to capture intra-neighborhood spatial variability yet coarse enough to support meaningful regionalization. Based on this resolution, we generated a $381 \times 434$ raster data for each input feature. Based on the semivariogram of each input feature (SM Fig. 1 of Supplementary Material), we determined a series of geo-thresholds for the regionalization based on spatially constrained SOM between 0.3 km and 3 km. With these geo-thresholds, we set the number of SOM neurons as between $72\times 72$ and $7\times 7$. Then, through Step 1 ***Embedding***, the rasterized input features (Fig. 3a) were stacked and transformed through the ***Embedding*** process using an autoencoder, which converted the original six-dimensional inputs into 12-dimensional representations that capture both individual feature characteristics and inter-feature interactions (SM Fig. 3 and SM Fig. 4 of Supplementary Material). The output of ***Embedding*** step was a $12 \times 381 \times 434$ multidimensional map, where each cell of the grid was represented by a $1 \times 12$ embedded vector of input features. Meanwhile, the locations and initial states of these SOM neurons are determined through a spatial partition of the study area based on the number of SOM neurons.

With the multidimensional map and the initial states of SOM neurons, the ***clustering*** step configured the SOM with different combinations of the number of SOM neurons and geo-thresholds. Each configuration was assessed using quantization error (QE) and geographic error (GE), as detailed in the Methods section, with the corresponding evaluation results provided in the Supplementary Materials. Among the SOM configurations, we finally trained an SOM model with $72\times 72$ neurons and a geo-threshold of 0.3 km, which achieved the lowest QE of 0.1948 and the lowest GE of 0.1778, indicating a balanced performance in preserving data fidelity and geographic coherence.

The trained spatially constrained SOM model then assigned each grid cell in the study area with the weights of their BMUs (SM Fig. 6 of Supplementary Material), and the output was used in Step 3: ***Refining***, where spatial partition and a region-growing algorithm were applied to generate the final regionalization of the case area (Fig. 3). Specifically, the spatial partition process aggregated the spatially constrained SOM outputs into 500 initial regions, then the region-growing algorithm merged these regions into 253 final regions that respect both feature similarity and geographic proximity. Compared with traditional methods such as SKATER, REDCAP, and standard GeoSOM (SM Fig. 7 of Supplementary Material), RepSC-SOM produces regions with greater spatial compactness and reduced fragmentation, making it more suitable for planning applications.

### Comparison of regionalization outputs and standard planning units

**Fig. 4:**
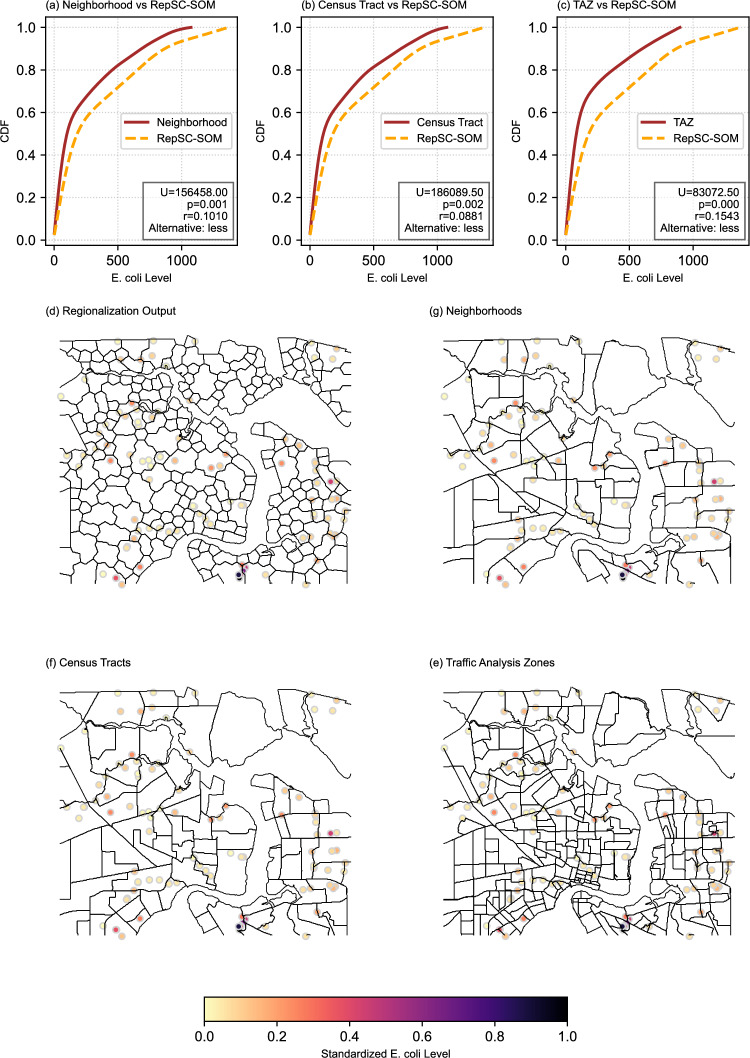
Comparison of regionalization outputs. Subfigures (**a**–**c**) show the cumulative distribution functions (CDFs) of cross-region differences in E. coli concentrations between the RepSC-SOM output and (**a**) neighborhoods, (**b**) census tracts, and (**c**) traffic analysis zones (TAZ). Subfigures (**d**–**g**) visualize the corresponding spatial delineations of regions: (**d**) RepSC-SOM, (**e**) neighborhoods, (**f**) census tracts, and (**g**) TAZ.

We compared the RepSC-SOM regionalization output with three standard planning units: census tracts, traffic analysis zones (TAZ), and neighborhoods (Fig. 4). Before comparison, we examined the size distributions of planning units generated by RepSC-SOM and the three standard units (SM Fig. 8 of the Supplementary Material). The results show that RepSC-SOM regions are generally smaller than census tracts and neighborhoods, but larger than TAZs. The comparison was based on the pairwise difference of E. coli concentrations between sample locations within each region and those in neighboring regions. The unit of E. coli concentration was measured in colony-forming units per 100mL (CFU/100mL) or the most probable number per 100mL (MPN/100mL)^57–60^.

Based on the Mann–Whitney U-Test, the RepSC-SOM regionalization exhibited significantly higher pairwise differences compared to census tracts ($p=0.002, r = 0.088$), TAZ ($p<0.001, r = 0.154$), and neighborhoods ($p=0.001, r = 0.101$), indicating a stronger ability to delineate areas with high water contamination. Across all comparisons, the average pairwise difference (after removing outliers based on Interquartile Range, IQR) for RepSC-SOM was 223.3 per 100 mL, whereas the corresponding values for census tracts, TAZ, and neighborhoods were 153.95 per 100 mL, 117.74 per 100 mL, and 145.26 per 100 mL, respectively. Furthermore, this comparative advantage persists across nine alternative configurations derived from our sensitivity analyses, with all yielding statistically significant improvements over administrative units (Supplementary Material Table 2). These results demonstrate that our framework can more accurately capture spatial heterogeneity in water quality, highlighting regions of potential concern for targeted interventions.

## Discussion

Previous regionalization efforts have rarely addressed the persistent mismatch between standard planning units, i.e., administrative boundaries or census divisions, and the spatial heterogeneity of climate and disaster risks. This mismatch can obscure where adaptation demands are concentrated, causing planning interventions to diverge from local risk patterns and reducing the effectiveness of adaptation strategies. Moreover, existing methods are unlikely to provide flexibility in integrating high-resolution local data, contextual knowledge, and governance priorities into the regionalization process, constraining their applicability to real-world urban governance. Our study addressed this gap by developing a demand-oriented, data-driven approach that advances both methodological design and planning relevance. In our case study, RepSC-SOM produced significantly greater differentiation in flooding risks (measured by E. coli concentrations, 223.30 per 100mL) than census tracts (153.95 per 100mL, $p=0.002,r=0.088$), TAZ (117.74 per 100mL, $p<0.001, r=0.154$), and neighborhoods (145.26 per 100mL, $p=0.001,r=0.101$), demonstrating its stronger capacity to capture local risk patterns.

Our study contributes both insights for urban governance and methodological innovations in regionalization. First, the performance of our data-driven regionalization demonstrates the limitations that administrative boundaries or census divisions may not reflect the spatial heterogeneity in climate risk, underscoring the value of locally informed regionalization for targeted interventions and data-informed urban governance^1,10,15,16^. Our findings can also be connected to MAUP, as the observed differences between regions derived from RepSC-SOM and administrative units illustrate how boundary definitions can influence analytical outcomes^12,33^. These results highlight the importance of scale dependence and spatial aggregation in regionalization. By deriving regions through a data-driven workflow guided by the topics of interest (such as water quality issues), our framework provides an alternative that helps mitigate such scale and zoning effects. Second, by enabling planners to select input features aligned with adaptation priorities and set geo-thresholds based on the spatial statistics of the data, our approach is flexible enough to support adaptation planning for other hazards, such as extreme heat or air pollution, and adapted to cities with varying built and socioeconomic environments^21,24^. This adaptability is essential in light of the dynamic nature of climate risks and the need for planning methods that remain responsive to shifting vulnerabilities and emerging challenges^22^. Third, advancing GeoSOM^44,45^ and clustering methods that are purely spatially constrained^18,32^, our design integrates deep representation learning with topology-preserving clustering and spatial refinement. This enables the identification of complex cross-variable interactions and the generation of regions that reflect multidimensional risk profiles while maintaining geographic coherence. The refining process in RepSC-SOM further ensures that the generated regions are analytically robust and operationally practical to planning, resource allocation, and risk communication, bridging the gap between technical rigor and governance demands in climate resilience planning.

Our study still has limitations that need to be considered. First, our analysis focused on a single city, Jacksonville, Florida, which only represents its own unique characteristics. Second, the input features in this study were selected based on data availability and their relevance to flood-induced water contamination. However, other factors, such as informal drainage systems or real-time hydrological dynamics, may also influence the spatial distribution of water contamination levels and the usability of our regionalization output. Third, RepSC-SOM integrates high-resolution local data, relying on the quality and completeness of available datasets. Missing or inconsistent data may affect the delineation of regions.

Future work should apply the RepSC-SOM framework to cases with diverse contexts and different disaster types to validate the framework’s capacity regarding adaptability, robustness, and effectiveness in capturing local hazard risk patterns for targeted adaptation planning. With diverse contexts and datasets in future work, we will assess the spatial dependence on validation data to ensure the statistical validity of between-region comparisons further. Future work can explore the incorporation of additional environmental and infrastructural variables to capture complex hazard dynamics better and improve the representativeness of generated regions. Future research will investigate methods for handling incomplete or uncertain data, including data imputation and robustness testing, to enhance the reliability of the framework and its applicability to evidence-based decision-making across diverse urban contexts. Future work will also explore integrating human-in-the-loop mechanisms that allow planners to interactively adjust model parameters or regionalization outcomes, combining domain expertise with data-driven insights to support more context-sensitive planning decisions.

## Conclusion

This study demonstrates that demand-oriented regionalization reflects local nuanced conditions more accurately than existing standard planning units, such as administrative boundaries or census divisions. The proposed framework, RepSC-SOM, supports urban governance and climate adaptation planning by providing data-informed units for analysis and intervention that can capture the distinctive patterns of local risks. Units created by RepSC-SOM can complement census-based or neighborhood boundaries that are more fixed by detecting risk patterns at finer spatial scales to bridge gaps between existing planning boundaries, or at coarser scales when data sensitivity is a concern. The framework is transparent, ensuring that regional boundaries are produced through a reproducible and auditable process rather than subjective or politically inherited zoning, thereby supporting more targeted adaptation planning decisions. By offering a flexible regionalization strategy that adapts to planning objectives and data constraints, RepSC-SOM extends the analytical toolkit available for spatially targeted adaptation planning.

## Methods

### Data preparation

We selected a study area within Jacksonville, Florida, a coastal city highly exposed to flood hazards. A uniform $50\times 50$m grid was generated for regionalization, with rivers and open water masked. In order to adapt to coastal floods, six characteristics reflecting flood exposure and local socio-economic conditions were used as inputs. All these features were converted to raster layers aligned with the grid and normalized to $[0,1]$ using Min–Max scaling.

- **Estimated inundation level in meters above ground level (m AGL)** based on the simulation of floodwater under a Category 5 hurricane scenario^61^.
- **Density of septic tanks** per grid cell, serving as a proxy for sanitation vulnerability during flood events^62^.
- **Percentile of within-cell average property values** (U.S. dollars per acre among all grid cells in the study area^63^.
- **Proportion of built-up and transportation areas** among all types of land cover within the grid cell^64^.
- **Proportion of impervious land areas(%)** within the grid cells^65^.
- **Population density** in the grid cells based on Data for Good at Meta^66^.

The geostatistics of the input data provide the basis for determining the geo-threshold $\delta$ used in the three-step regionalization described in the next section. Specifically, this threshold—derived from the features’ spatial autocorrelation—can define the maximum allowable separation between neighboring grid cells. Its lower and upper bounds were set from the range of semivariogram values^67^, corresponding to the distances where spatial autocorrelation stabilized. Based on the semivariograms in this study (SM Fig.1), the lower and upper boundaries were set to 0.15 km and 0.3 km, respectively. Both the geo-thresholds and input feature data were used in the three-step regionalization process.

### Three steps of RepSC-SOM

The parameters determined in the three-step RepSC-SOM are summarized in Table 1, along with the methods used for their determination.

**Table 1:** Summary of parameters involved in RepSC-SOM and their determination methods.

**Parameter**	**Value**	**Determination Method**
Data resolution	50 m $\times$ 50 m	Research question (neighborhood scale)
Geo-threshold range	0.3–3 km	Semivariogram analysis
SOM neurons range	7$\times$7 to 72$\times$72	Derived from geo-threshold range
Embedding dimensions	12	Autoencoder optimization
Selected geo-threshold	0.3 km	Lowest QE and GE
Selected SOM neurons	72$\times$72	Lowest QE and GE
Count of initial regions (*N*)	500	Spatial partition
$\tau$	0.3	Sensitivity analysis (SM Table 1)
Area_max_	1,600 grid cells	Sensitivity analysis (SM Table 1)
Convex_min_	0.8	Sensitivity analysis (SM Table 1)
Count of final regions	253	Output of region-growing

#### Step 1: embedding

The input features were embedded into a higher-dimensional latent representation using an autoencoder^52^, to capture complete information about the features and their interactions. The autoencoder employed an encoder to project the multidimensional input features into a latent-space representation of higher or lower dimensionality, followed by a decoder to reconstruct the original features from this representation. We trained a series of autoencoders with varying latent space dimensionalities to identify the one that minimized the reconstruction error while maximizing the coefficient of determination ($R^2$) between the input features and the reconstructed outputs. Based on the test results (SM Fig. 2) and the elbow criterion, we selected the autoencoder that embedded the input features into a 12-dimensional representation, achieving a reconstruction error of 0.024 and a high $R^2$ of 0.976, indicating strong fidelity to the original feature space.

Before inputting the embeddings into the ***Clustering*** process, initialization was required, including specifying the number of neurons in the SOM and their initial states. The number of neurons in the SOM was determined by the geo-threshold and scale of the study area, ensuring that the neighborhood ranges of all neurons collectively covered the entire region.

$$N = \left\lceil {\frac{{\max \left\{ {Width_{{vertical}} ,Width_{{horizontal}} } \right\}}}{\delta }} \right\rceil$$

where the square of *N*, i.e., $N^2$, is the number of SOM neurons, $Width_{vertical}$ and $Width_{horizontal}$ are the vertical and horizontal widths of the study region, $\delta$ is the geo-threshold, and $\lceil \cdot \rceil$ is the ceiling function. In particular, this calculation determined the minimum number of SOM neurons required for a given geo-threshold, and the total number of neurons can exceed this minimum, allowing multiple combinations of neuron numbers and thresholds. In this study, the candidate geo-thresholds were $\{0.3, 0.6, 0.9, 1.2, 1.5, 1.8, 2.1, 2.4, 2.7, 3\}\,\text {km}$, and the candidate counts of the SOM neurons were $\{72, 36, 24, 18, 14, 12, 10, 9, 8, 7\}$, forming a total of 55 possible SOM configurations, e.g., a threshold of 0.3 km with 72 neurons.

With the number of SOM neurons determined, we also set their initial states, including their locations and weights. To ensure full coverage of the study area at the start of ***Clustering***, we used spatial partitioning based on the ***Embedding*** output, taking the geometric centroids of the partitions as neuron locations. In this study, partitions were generated using simple linear iterative clustering (SLIC)^68^ via the scikit-image package^69^. If the number of partitions was fewer than the expected SOM neurons, additional locations were randomly sampled from the study area; if more, a subset of partition centroids was randomly selected as initial neuron locations. After specifying the locations, the initial weights of the SOM neurons were assigned to match those of the nearest grid cells in the study area. This initialization strategy for SOM neurons can ensure full spatial coverage and balanced representation across the study area, reducing the dependence on random placement and providing a geographically coherent starting point for subsequent learning.

#### Step 2: clustering

The ***Clustering*** step involved training a spatially constrained SOM using a two-phase Best Matching Unit (BMU) selection strategy, followed by iterative weight updates. Each grid cell in the study area was represented as $(\textbf{x}_i, \textbf{g}_i)$, where $\textbf{x}_i \in \mathbb {R}^d$ was the embedded feature vector and $\textbf{g}_i \in \mathbb {R}^2$ its geographic coordinates. Each SOM neuron $k \in \{1,\dots ,S\}$ was defined by a weight vector $\textbf{w}_k \in \mathbb {R}^d$ in the embedded feature space and geographic coordinates $\textbf{c}_k \in \mathbb {R}^2$, determined during initialization.

The first phase of BMU selection was ***Geographic filtering***. Here, candidate SOM neurons were first identified within a geo-threshold $\delta$ around each grid cell, where distances were computed using the Haversine formula between the cell’s coordinates $\textbf{g}_i$ and the neuron’s coordinates $\textbf{c}_k$.

$$\mathcal {C}_i = \{ k \mid \text {Haversine}(\textbf{g}_i, \textbf{c}_k) < \delta \}.$$

If no neurons fell within this threshold $\delta$, all SOM neurons were included as candidates for BMU selection, i.e., $\mathcal {C}_i = \{1, \dots , S\}$.

Within the candidate SOM neurons $\mathcal {C}_i$ after the first phase, the second phase, ***Feature-based Matching***, selected the final BMU for each grid cell as the SOM neuron with the minimum Euclidean distance between the grid cell’s feature vector $\textbf{x}_i$ and the weights of the candidate neurons $\textbf{w}_k$:

$$k^* = \arg \min _{k \in \mathcal {C}_i} \Vert \textbf{x}_i - \textbf{w}_k\Vert _2.$$

Once the BMU $k^*$ was identified for each grid cell $(\textbf{x}_i, \textbf{g}_i)$, the SOM model updated the weights of BMUs and their neighboring neurons. Specifically, at iteration $t$, the weight vector of the SOM neuron $k$ was updated as:

$$\textbf{w}_k(t+1) = \textbf{w}_k(t) + \alpha (t)\, h_{k^*k}(t)\, (\textbf{x}_i - \textbf{w}_k(t)),$$

where $\alpha (t)$ is the learning rate and $h_{k^*k}(t)$ is the neighborhood function measuring the influence of the BMU on neuron $k$. A Gaussian kernel was used:

$$h_{k^*k}(t) = \exp \left( -\frac{\Vert r_{k^*} - r_k \Vert ^2}{2\sigma (t)^2}\right) ,$$

with $r_k$ denoting the 2D grid index of neuron $k$ and $\sigma (t)$ the neighborhood radius. Both $\alpha (t)$ and $\sigma (t)$ decreased over iterations, allowing the SOM model to capture broad global structure during early training and refine local details in later stages. This ***Clustering*** process can ensure that grid cells closer to the SOM neurons were updated more intensively, producing clustering results that preserved both feature similarity and spatial proximity.

The SOM model training in the ***Clustering*** process was evaluated using Quantization Error (QE) and Geographic Error (GE). These metrics can capture how well the spatially constrained SOM model preserved the similarity of embedded features and the geographic structure of the study area. Specifically, QE was the average Euclidean distance between each grid cell’s feature vector $\textbf{x}_i$ and the weight vector of its BMU, $\textbf{w}_{k^*}$. Lower QE values can indicate better representation of the data of the embedded features by the spatially constrained SOM model.

$$\text {QE} = \frac{1}{N} \sum _{i=1}^N \left\| \textbf{x}_i - \textbf{w}_{k^*} \right\| _2,$$

where $k^* = \arg \min _k \left\| \textbf{x}_i - \textbf{w}_k \right\| _2$, and $N$ is the total number of grid cells.

GE quantified the spatial distortion introduced during BMU assignment and was computed as the average Haversine distance between the geographic coordinate of each data point $\textbf{g}_i$ and that of its BMU $\textbf{c}_{k^*}$. Lower GE values can imply that the spatial structure of the study area was well preserved in the resulting clusters.

$$\text {GE} = \frac{1}{N} \sum _{i=1}^N \text {Haversine}(\textbf{g}_i, \textbf{c}_{k^*}).$$

In this study, we tested 55 combinations of geo-thresholds and SOM neuron counts generated in the ***Embedding*** step (QE and GE are shown in SM Fig. 5). We eventually selected a geo-threshold of 0.3 km and 72 SOM neurons, based on their overall low QE and GE. The spatially constrained SOM model with this configuration was adopted, assigning each grid cell the feature weights of its BMU, and this output was the basis for the next ***Refining*** step.

#### Step 3: refining

The ***Refining*** step began with a spatial partition of the ***Clustering*** output, aiming to base the refinement on larger regions rather than individual grid cells, since cell-level merging or grouping can be relatively unstable and less robust. The ***Refining*** step was controlled by the following indicators as the configuration: the number of spatial partitions $P$, the threshold for deciding region merging $\tau$, the maximum size of each final region $Area_{\max }$, and the convexity of the final regions $Convex_{\min }$. First, adopting the same procedure as in the ***Embedding*** step, we applied SLIC with the ***Clustering*** output for spatial partition, generating regions $s_i$ with the count of $P$. Each region was assigned a mean feature vector $\textbf{f}_i$ among the inside grid cells and area $Area_i$. Neighboring regions $s_j$ can be merged with $s_i$ if the Euclidean distance of their feature vectors satisfied

$$\Vert \textbf{f}_i - \textbf{f}_j\Vert _2 < \tau ,$$

and the combined area did not exceed the maximum allowed size

$$Area_i + Area_j \le Area_{\max }.$$

Convexity of a merged region $S_{merged}$ was defined as

$$Convex = \frac{\text {Area}(S_{merged})}{\text {Area}(\text {ConvexHull}(S_{merged}))},$$

and merging was constrained such that $Convex \ge Convex_{\min }$ to prevent regions with non-convex shapes. This iterative region-growing process continued until merging between regions could be conducted to satisfy both the feature similarity and spatial constraints, producing a final set of coherent regions. The output of the region-growing process was considered the final regionalization output.

The quality of the ***Refining*** output was evaluated in terms of compactness and homogeneity. **Compactness** of each region was calculated as

$$Compact_k = \frac{4 \pi \, Area_k}{Perim_k^2},$$

where $Area_k$ was the area (number of grid cells) of region $k$ and $Perim_k$ was its perimeter computed from the outer boundaries of the region. Compactness values closer to 1 indicate more compact shapes, although aliasing effects may yield values slightly above 1^70^. The overall compactness score for the regionalization output was taken as the mean over all regions:

$$Compact = \frac{1}{N} \sum _{k=1}^{N} Compact_k,$$

where $N$ was the total number of regions in the final regionalization output. **Homogeneity** of a region was measured by the variance of the feature vector values of grid cells within the region. For a region $k$ with feature vectors $\textbf{f}_i$ for all cells $i \in k$, the variance was computed as

$$H_k = {\left\{ \begin{array}{ll} \textrm{Var}(\{\textbf{f}_i\}) & \text {if 1-dimension features},\\ \frac{1}{d} \sum _{j=1}^{d} \textrm{Var}(\{f_{i,j}\}) & \text {if { d}-dimensional features}, \end{array}\right. }$$

and the overall homogeneity score of the regionalization was the mean variance across all regions:

$$H = \frac{1}{N} \sum _{k=1}^{N} H_k.$$

A higher compactness indicated more regularly shaped, spatially coherent regions, whereas a lower homogeneity score indicates more uniform feature values within regions.

In this study, we tested a series of configurations for the ***Refining*** process, and the twenty best-performing configurations in terms of compactness and homogeneity scores were reported in SM Table 1. Accordingly, we set $P$ as 500, $\tau$ as 0.3, $Area_{\max }$ as 1,600 grid cells, and $Convex_{\min }$ as 0.8, making the regionalization output have a compactness level of 1.13 and a homogeneity level of 0.02.

### Final result evaluation

The regionalization output was evaluated by its ability to characterize the water quality patterns in our study area. The water quality is represented by E. coli concentration at a set of measurement locations, where the data was collected by the US Geological Survey (USGS) and the Florida Department of Environmental Protection (FDEP)^57–60^. Each location was assigned to the region generated by a given regionalization method (e.g., our RepSC-SOM or census tracts), and additionally, each location was associated with all neighboring (adjacent) regions of its assigned region. Regions without any measurement locations were excluded from the evaluation.

For each region $R_i$ with *n* locations of water quality data $Q_i = \{Q_{i1}, Q_{i2}, \dots , Q_{in}\}$ and each neighboring region $R_j$ with *m* locations $Q_j =\{Q_{j1}, Q_{j2}, \dots , Q_{jm}\}$, the pairwise differences in water quality data were computed as

$$D_{ij} = \{|Q_{ik} - Q_{jl}| : k=1,\dots ,n; \ l=1,\dots ,m\}.$$

All pairwise differences for a given regionalization method were aggregated across all regions with measurements:

$$D = \bigcup _{i,j} D_{ij}.$$

To compare the effectiveness of different regionalization methods in highlighting areas with water quality issues, the distributions of pairwise differences $D$ from each method were statistically compared using the Mann–Whitney U-Test^71^. A regionalization was considered better if it produced significantly higher pairwise differences (p-value $< 0.05$), indicating that it can better separate areas with distinct water quality levels and thus more effectively identify areas of concern for climate adaptation planning.

## Supplementary Information

Supplementary Information.

## Data Availability

The code developed by S.M.N. for this study is publicly available on GitHub (https://github.com/mobinanoorany/RepSC-SOM). The input data for regionalization is publicly available from Zachry et al. (https://doi.org/10.1175/WCAS-D-14-00049.1), Florida Department of Health (https://www.floridahealth.gov/environmental-health/), University of Florida GeoPlan Center (https://fgdl.org/), National Oceanic and Atmospheric Administration (NOAA) Office for Coastal Management (https://coast.noaa.gov/), Florida Department of Environmental Protection (https://geodata.dep.state.fl.us/), and Meta (https://data.humdata.org/dataset/united-states-high-resolution-population-density-maps-demographic-estimates). The water quality data were downloaded from STORET (https://prodapps.dep.state.fl.us/dear-spa/public/welcome) and the WIN (https://prodenv.dep.state.fl.us/DearWin/public/welcomeGeneralPublic) databases of the Florida Department of Environmental Protection. The National Park Service Water Resources Division, Florida Department of Environmental Protection, City of Jacksonville, St. Johns RiverWater Management District, Florida Fish and Wildlife Research Institute, Florida Lakewatch, and Jacksonville Electric Authority contributed data to STORET. Florida Department of Environmental Protection, City of Jacksonville, St. Johns River Water Management District, and Florida Lakewatch contributed to WIN. The generated experimental data can be obtained from the corresponding author upon reasonable request.
