# Supplementary material for: Demand-oriented regionalization with local data
Source: Sci Rep. 2026 Apr 20;16:18241. doi: 10.1038/s41598-026-47764-4 (PMC13260847; doi:10.1038/s41598-026-47764-4)
Supplement: Supplementary file 1 — Supplementary Information. [file 41598_2026_47764_MOESM1_ESM.pdf]

Demand-Oriented Data-Driven Regionalization for Climate Adaptation Planning (Supplementary Material)

Seyedeh Mobina Noorani[1,2*], Shangde Gao[1,3], Changjie Chen[1,3], Karla Saldaña Ochoa[1**]

[1]College of Design, Construction and Planning, University of Florida, Gainesville, FL 32611 USA

[2]Department of Electrical and Computer Engineering, University of Florida, Gainesville, FL 32611 USA

[3]Florida Institute for Built Environment Resilience (FIBER), University of Florida, Gainesville, FL 32611 USA

[*]s.noorani@ufl.edu [**]ksaldanaochoa@ufl.edu

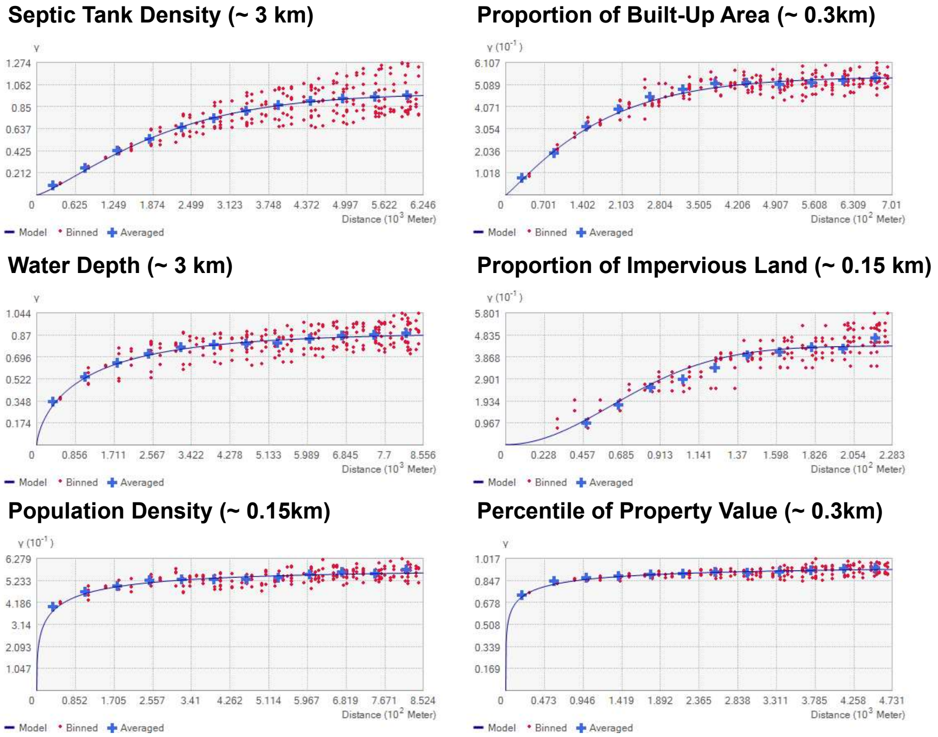

Figure 1: Semivariograms for each input feature

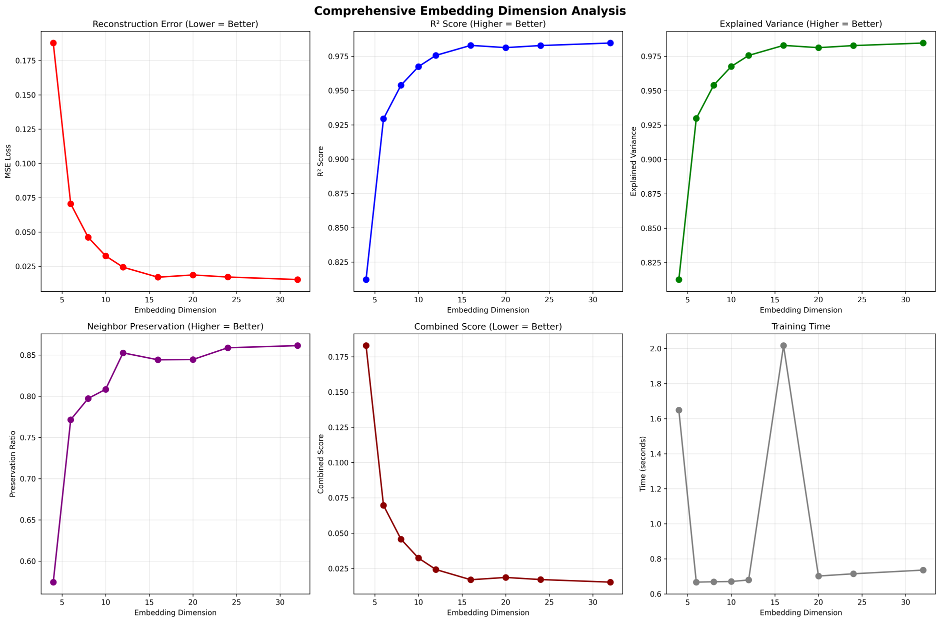

Figure 2: Configurations tested for the ***Embedding*** process with different number of output dimensions for autoencoder

Feature Contribution to Embedded Features (Correlation Coefficients)

Latent Feature	Septic Tank Density	Inundation Level (m AGL)	Built-up and Transportation Proportion	Impervious Land Proportion (%)	Property Value Percentile	Population Density
**Latent 1**	0.714	-0.413	0.197	0.140	-0.110	0.651
**Latent 2**	0.489	0.279	0.208	0.249	-0.523	0.440
**Latent 3**	-0.002	0.165	-0.776	-0.623	0.090	0.310
**Latent 4**	-0.292	-0.188	0.395	0.900	0.147	0.197
**Latent 5**	0.125	-0.251	0.223	0.378	0.472	0.739
**Latent 6**	0.080	-0.919	0.367	0.360	-0.251	0.373
**Latent 7**	-0.239	0.099	-0.262	-0.241	0.835	-0.022
**Latent 8**	-0.350	0.524	-0.690	-0.461	-0.279	-0.104
**Latent 9**	-0.157	0.416	-0.550	0.191	0.351	0.193
**Latent 10**	0.099	-0.056	-0.657	-0.004	-0.008	-0.441
**Latent 11**	0.592	0.247	-0.695	-0.537	0.119	-0.124
**Latent 12**	0.305	0.548	-0.183	0.126	0.664	-0.335

Figure 3: Correlation coefficients between input features and embedding dimensions

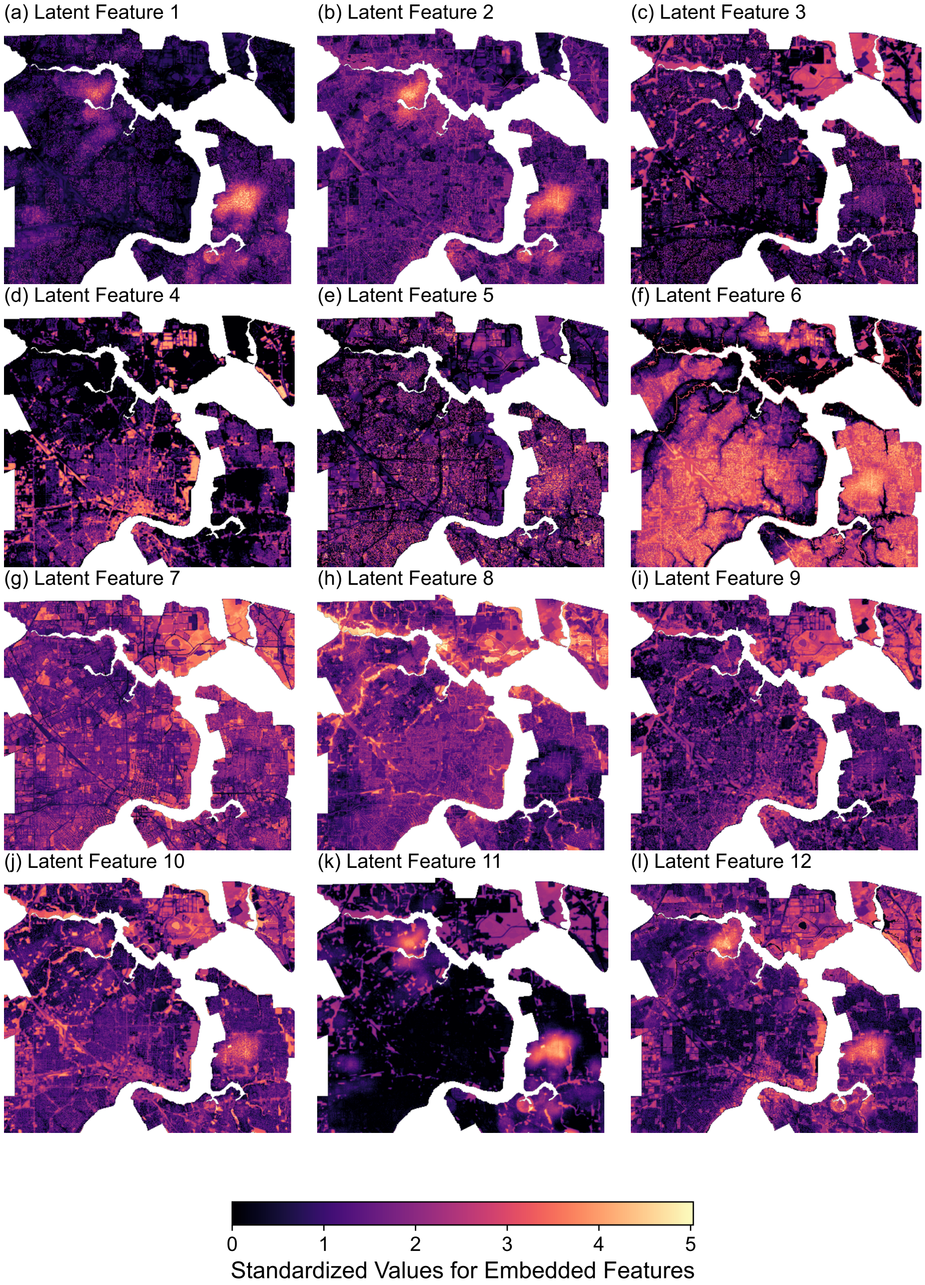

Figure 4: Spatial patterns of each latent dimension after ***Embedding*** step

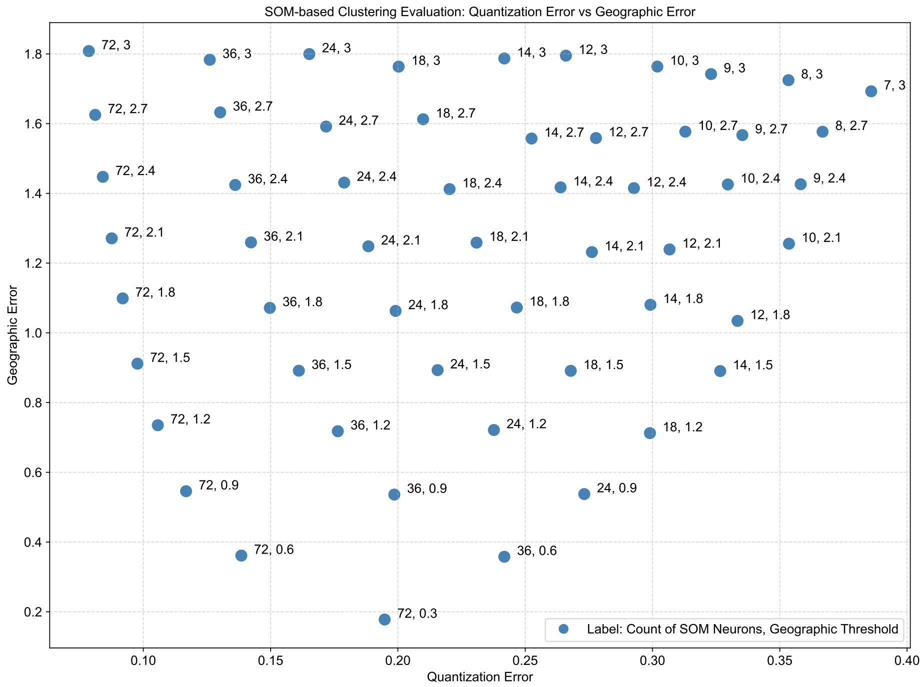

Figure 5: SOM configurations tested for the ***Clustering*** process

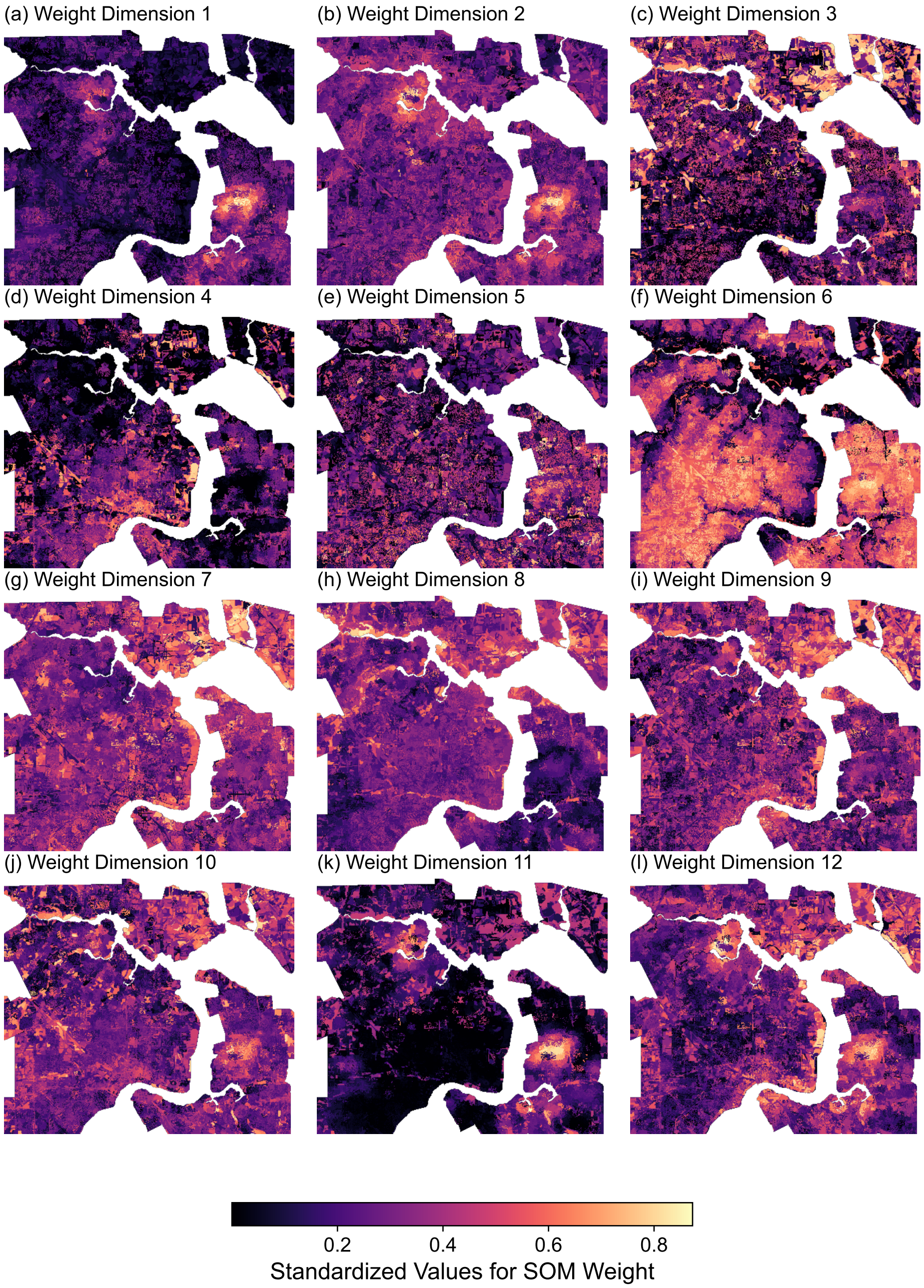

Figure 6: Spatial patterns of each dimension of SOM after ***Clustering*** step

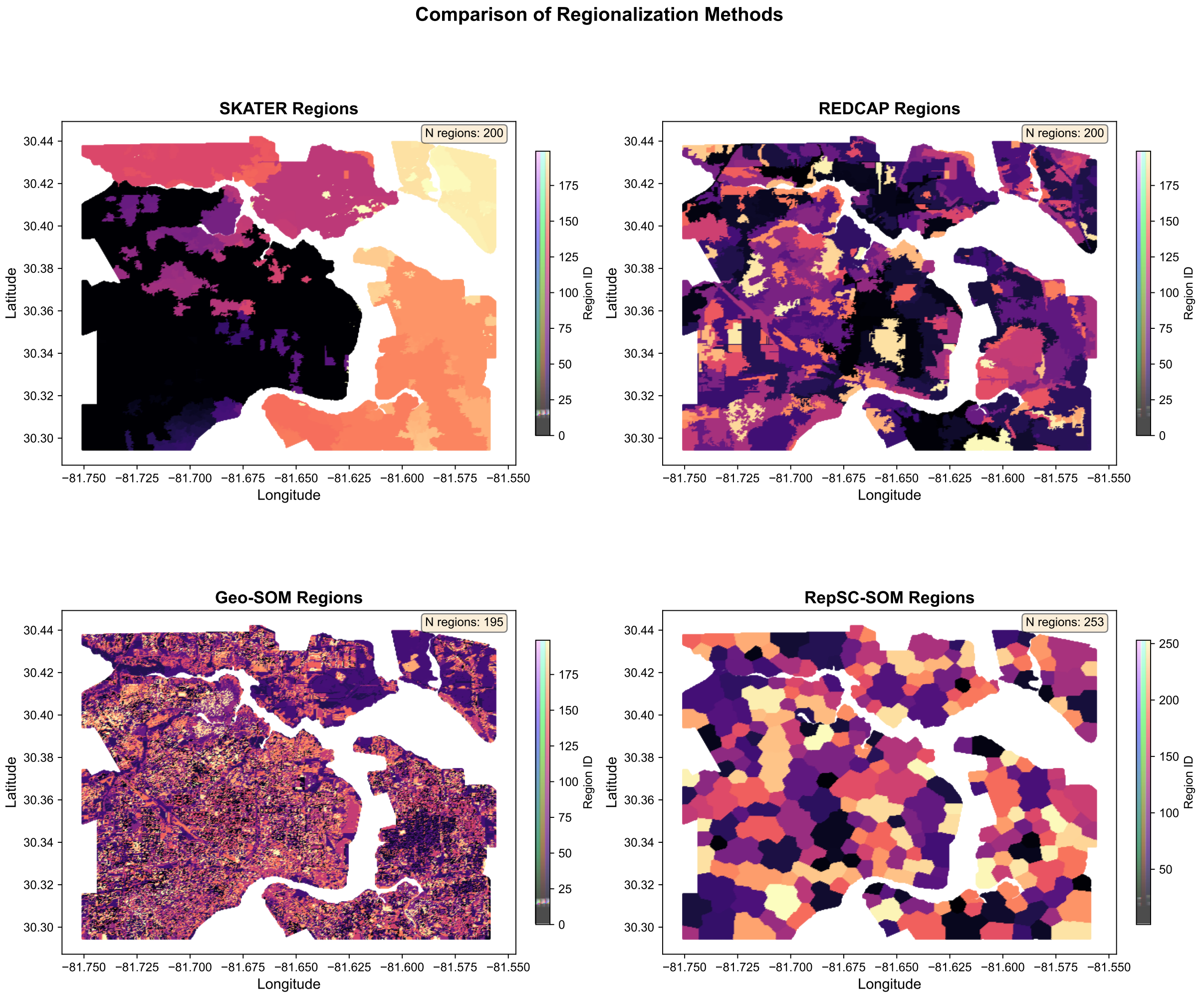

Figure 7: Regionalization outputs of traditional methods: SKATER, REDCAP, and standard Geo-SOM, and our RepSC-SOM outputs

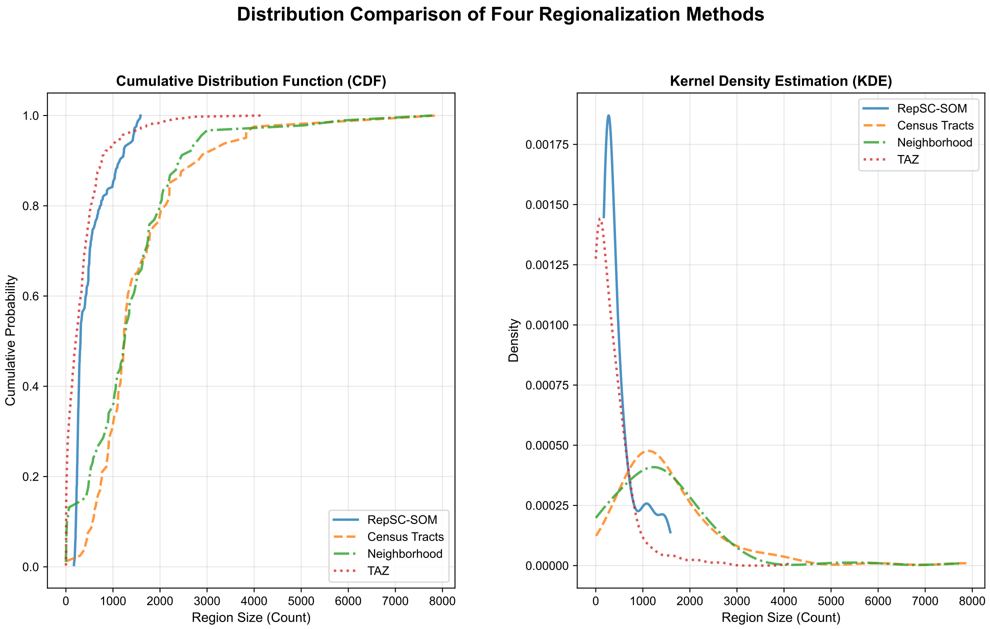

Method	Count	Mean	Median	Std Dev
RepSC-SOM	253	501.22	310.00	375.48
Census Tracts	81	1565.53	1238.00	1191.70
Neighborhood	91	1393.49	1251.00	1191.94
TAZ	369	343.65	207.00	463.48

Figure 8: Statistical distributions of the sizes of regions generated by RepSC-SOM, census tracts, neighborhoods, and traffic analysis zones (TAZ)

Table 1: Configurations tested for the ***Refining*** process

Count of spatial partition	Threshold for merging	Max count of regions	Convexity	Compactness	Homogeneity
500	0.3	1600	0.8	1.1262	0.0200
500	0.2	1600	0.8	1.1293	0.0195
500	0.1	1600	0.7	1.1231	0.0192
500	0.1	1600	0.6	1.1175	0.0192
500	0.2	900	0.8	1.1225	0.0191
500	0.1	1600	0.8	1.1468	0.0186
500	0.3	900	0.8	1.1088	0.0192
500	0.1	900	0.8	1.1462	0.0185
500	0.1	900	0.7	1.1197	0.0188
500	0.1	900	0.6	1.1166	0.0188
500	0.2	1600	0.7	1.0356	0.0202
500	0.1	400	0.6	1.1546	0.0179
500	0.1	400	0.7	1.1546	0.0179
500	0.1	400	0.8	1.1546	0.0179
500	0.2	400	0.6	1.1546	0.0179
500	0.2	400	0.7	1.1546	0.0179
500	0.2	400	0.8	1.1546	0.0179
500	0.3	400	0.6	1.1546	0.0179
500	0.3	400	0.7	1.1546	0.0179
500	0.3	400	0.8	1.1546	0.0179

Table 2: Mann–Whitney U-Test comparison of alternative RepSC-SOM configurations against administrative units.

N	τ	$\mathbf{Area_{max}}$	$\mathbf{Convex_{min}}$	**Comparison Unit**	**U**	**p**
500	0.3	1600	0.8	Neighborhood	156458.0	0.001**
				Census Tract	186089.5	0.002**
				TAZ	83072.5	0.000***
500	0.2	1600	0.8	Neighborhood	109263.5	0.002**
				Census Tract	129938.0	0.005**
				TAZ	58009.5	0.000***
500	0.1	1600	0.7	Neighborhood	96415.0	0.016*
				Census Tract	114685.5	0.035*
				TAZ	51133.5	0.000***
500	0.1	1600	0.6	Neighborhood	95095.5	0.015*
				Census Tract	113137.5	0.035*
				TAZ	50408.5	0.000***
500	0.2	900	0.8	Neighborhood	106380.5	0.002**
				Census Tract	126537.0	0.005**
				TAZ	56467.0	0.000***
500	0.1	1600	0.8	Neighborhood	85800.5	0.010**
				Census Tract	102052.0	0.023*
				TAZ	45538.0	0.000***
500	0.3	900	0.8	Neighborhood	138787.5	0.001**
				Census Tract	165112.0	0.002**
				TAZ	73611.5	0.000***
500	0.1	900	0.8	Neighborhood	84542.5	0.008**
				Census Tract	100566.0	0.018*
				TAZ	44854.0	0.000***
500	0.1	900	0.7	Neighborhood	95157.0	0.012*
				Census Tract	113199.5	0.028*
				TAZ	50449.5	0.000***
500	0.1	900	0.6	Neighborhood	92363.0	0.019*
				Census Tract	109874.5	0.041*
				TAZ	48958.5	0.000***

* $p < 0.05$, ** $p < 0.01$, *** $p < 0.001$
